# Exposure to foreign gut microbiota can facilitate rapid dietary shifts

**DOI:** 10.1038/s41598-021-96324-5

**Published:** 2021-08-18

**Authors:** C. Heys, A. M. Fisher, A. D. Dewhurst, Z. Lewis, A. Lizé

**Affiliations:** 1 Department of Evolution, Ecology and Behaviour, University of Liverpool, Liverpool, L69 7ZB UK; 2 School of Life Sciences and Education, Staffordshire University, Stoke-on-Trent, ST4 2RU UK; 3 School of Biological and Chemical Sciences, Queen Mary University of London, London, E1 4NS UK; 4 School of Life Sciences, University of Liverpool, Liverpool, L69 7ZB UK; 5 Laboratoire de Biologie des Organismes et Ecosystèmes Aquatiques (BOREA), Muséum National d’Histoire Naturelle (MNHN), Centre de Recherche et d’Enseignement sur les Systèmes Côtiers (CRESCO), Station Marine de Dinard, Dinard, France; 6 UMR CNRS 6553 ECOBIO, University of Rennes 1, 35042 Rennes, France

**Keywords:** Ecology, Evolution

## Abstract

Dietary niche is fundamental for determining species ecology; thus, a detailed understanding of what drives variation in dietary niche is vital for predicting ecological shifts and could have implications for species management. Gut microbiota can be important for determining an organism’s dietary preference, and therefore which food resources they are likely to exploit. Evidence for whether the composition of the gut microbiota is plastic in response to changes in diet is mixed. Also, the extent to which dietary preference can be changed following colonisation by new gut microbiota from different species is unknown. Here, we use *Drosophila spp.* to show that: (1) the composition of an individual’s gut microbiota can change in response to dietary changes, and (2) ingestion of foreign gut microbes can cause individuals to be attracted to food types they previously had a strong aversion to. Thus, we expose a mechanism for facilitating rapid shifts in dietary niche over short evolutionary timescales.

## Introduction

Dietary niche is fundamental in determining species ecology and the impact that organisms have on the landscape. As such, an informed understanding of dietary niche is important for successful species management strategies, such as conservation or pest control^1,2^. In addition, understanding the mechanisms that cause an organism’s dietary niche to change are important for predicting how species ecology will vary over time in response to environmental change. Rapid changes to the dietary niche of some organisms may create unexpected and potentially negative ecological challenges, such as increases in inter-species competition^3^ or the emergence of new pest species^4^. By contrast, shifting diet might be a way for a species/population to adapt and persist through rapid environmental changes such as reductions in food resources^5^. Therefore, identifying potential mechanisms for rapid dietary shifts is of importance.

It is well-known that genetically-fixed phenotypic traits which determine an organism’s diet can vary across generations, allowing individuals to adapt to alternative food sources over evolutionary timescales^6^. However, there are also non-fixed phenotypic traits which can determine an organism’s diet, allowing for dietary shifts to take place over much shorter timescales^7,8^. The gut microbiome plays an integral role in determining the ability of individuals to exploit certain food types^9^ and can vary within the lifetime of a host both autonomously and in response to environmental change^10^. As such, changes to the gut microbiome may represent a mechanism by which individuals can rapidly adapt to a novel dietary niche. However, the relationship between the gut microbiome and dietary niche requires further research to be fully understood.

The fruit fly *Drosophila sechellia* feeds on *Morinda citrifolia* (Tahitan noni fruit) which contains octanoic acid. Octanoic acid is fatal to other *Drosophila* species, including *Drosophila melanogaster*^11^. As such, *D. melanogaster* has a strong aversion to *M. citrifolia*^12^. In this study, we sequenced the gut microbiota of *D. sechellia* reared on standard fly medium (*N* = 12) and those reared on *M. citrifolia* (*N* = 12) to see whether the composition of the gut microbiota was associated with the consumption of *M. citrifolia*. In addition, to test whether acquisition of gut microbes from another species could alter food preference, we compared aversion to octanoic acid between *D. sechellia* (*N* = 100), *D. melanogaster* reared on standard fly medium (N = 100), and *D. melanogaster* that had been reared on food containing *D. sechellia* gut microbes for one (*N* = 101) and 10 (*N* = 97) generations.

## Results/discussion

### Dietary changes can drive variation in gut microbiota

**Figure 1:**
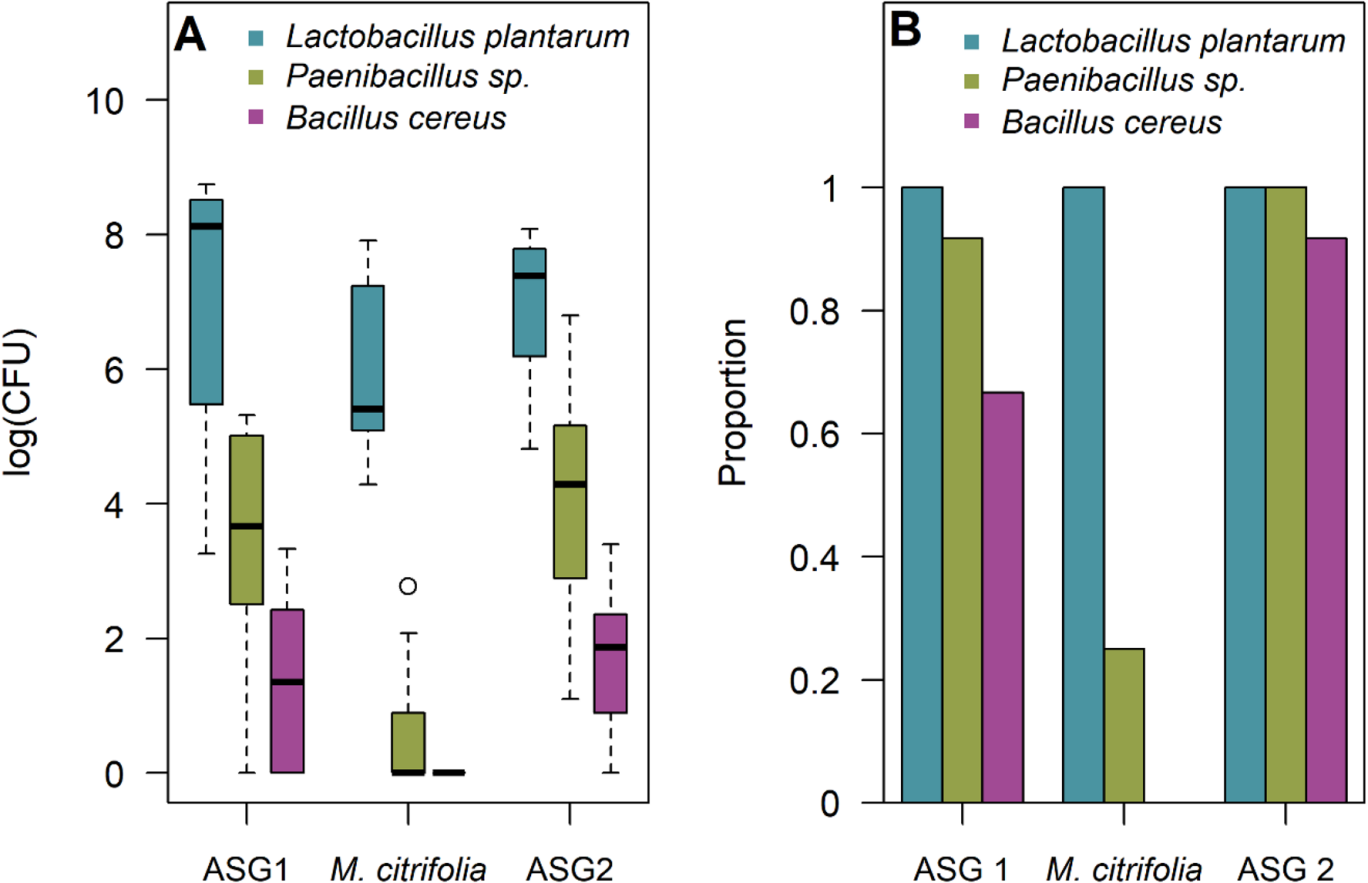
Variation in (**A**) the abundance (as measured by Colony Forming Units) of, and (**B**) the proportion of individuals harbouring *L. plantarum*, *Paenibacillus sp.*, and *Bacillus cereus* in the gut. Data is derived from gut dissections of adult *D. sechellia* that were fed on: (1) ASG for 1 week (ASG 1), (2) ASG for 1 week followed by *M. citrifolia* for 1 week (*M. citrifolia*), and (3) ASG for 1 week followed by *M. citrifolia* for 1 week before a final week of feeding on ASG (ASG 2).

The gut microbiota of *D. sechellia* reared on standard fly medium was characterised by three species of bacteria: *Lactobacillus plantarum*, *Paenibacillus sp.* and *Bacillus cereus* (Table 1, supplementary material). In our model analysing variation in bacterial abundance, the most parsimonious model included an interaction between bacterial species and diet as the only fixed effect (sex was omitted from the fixed effects), and the model fit was greatly improved by logging the response variable (abundance). The abundance of all three bacterial species was reduced when individuals were moved from an initial diet of ASG (ASG1) to a diet of *M. citrifolia* (Fig. 1A). However, this reduction was only significant for *Paenibacillus sp.* (*t* = − 4.17, *p* < 0.001) and was marginally non-significant for both *L. plantarum* (*t* = − 1.85, *p* = 0.078) and *B. cereus* (*t* = − 1.98, *p* = 0.061). Furthermore, prior to exposure to *M. citrifolia*, the gut microbiota of all individuals included at least two of these bacterial species, with 58.3% of individuals containing all three bacterial species. However, when *D. sechellia* were reared on a diet of *M. citrifolia*, the species richness of the gut microbiota was reduced such that in 75% of individuals, *L. plantarum* was the only bacterial species detected (Fig. 1B). Thus, our results suggest that feeding on *M. citrifolia* can reduce both the abundance and species richness of gut microbes in *D. sechellia*.

We detected no significant difference between the abundance of *L. plantarum* (*t* = − 0.30, *p* = 0.77), *Paenibacillus sp.* (*t* = 0.81, *p* = 0.43) or *B. cereus* (*t* = 0.50, *p* = 0.62) in individuals of the ASG1 and ASG2 treatment (Fig. 1A). Moreover, after returning to a diet of standard fly medium gut microbe species richness was restored such that the gut microbiota of all but one individual contained all three species of bacteria (Fig. 1B). Thus, our results also suggest that after feeding on *M.citrifolia*, gut microbe abundance and species richness in *D. sechellia* can be restored upon returning to a diet of standard fly medium.

Our findings are consistent with previous work showing reduced diversity of certain gut microbes in other fruit fly species which fed on acidic fruit^13^. Interestingly, despite undergoing a marginally non-significant reduction in abundance when their hosts fed on *M. citrifolia*, *L. plantarum* persisted in the guts of all the individuals used in this experiment, regardless of diet. The persistence of *L. plantarum* in the gut of *D. sechellia* could be explained by *L. plantarum* evolving to be resistant to the toxins contained in *M. citrifolia*. Additionally, certain species of *Drosophila* may have evolved to harbour *L. plantarum* in the gut as it incurs a fitness advantage. In other *Drosophila spp.*, *L. plantarum* is known to act as a growth promoter when resources are scarce^14^, and can provide protection against gut pathogens^15^. It may also be the case that *L. plantarum* acts as a detoxifying agent, allowing individuals to metabolise the otherwise toxic compounds found in certain plant species.

To-date, the cross-species evidence regarding the determinants of gut microbe community composition has been mixed. Several studies indicate that gut microbiota is phylogenetically constrained^9,16,17^, while others suggest that the gut microbiota can be plastic in response to host diet^18,19^. In this experiment, our data from *D. sechellia* shows that the gut microbiota can change in response to dietary variation. This variation could represent an adaptation that facilitates the exploitation of new food resources. Indeed, there is already evidence that larval *Drosophila spp.* switch to a cannibalistic diet when other food sources become scarce^20,21^, although whether this dietary switch in larvae is facilitated by changes to the gut microbiota is unknown. Our findings could have important implications for how individuals adapt to a changing environment, how their role in the ecosystem changes over time, and how these changes are facilitated by variation in gut microbiota. However, our result should be interpreted with caution, as we cannot say from our data whether the changes in the gut microbiota we observed serve an adaptive purpose, or are merely a by-product of variation in the resistance of different species of gut microbes to acidic food sources (in this case, *M. citrifolia*).

### Ingestion of foreign gut microbes can alter dietary preference

**Figure 2:**
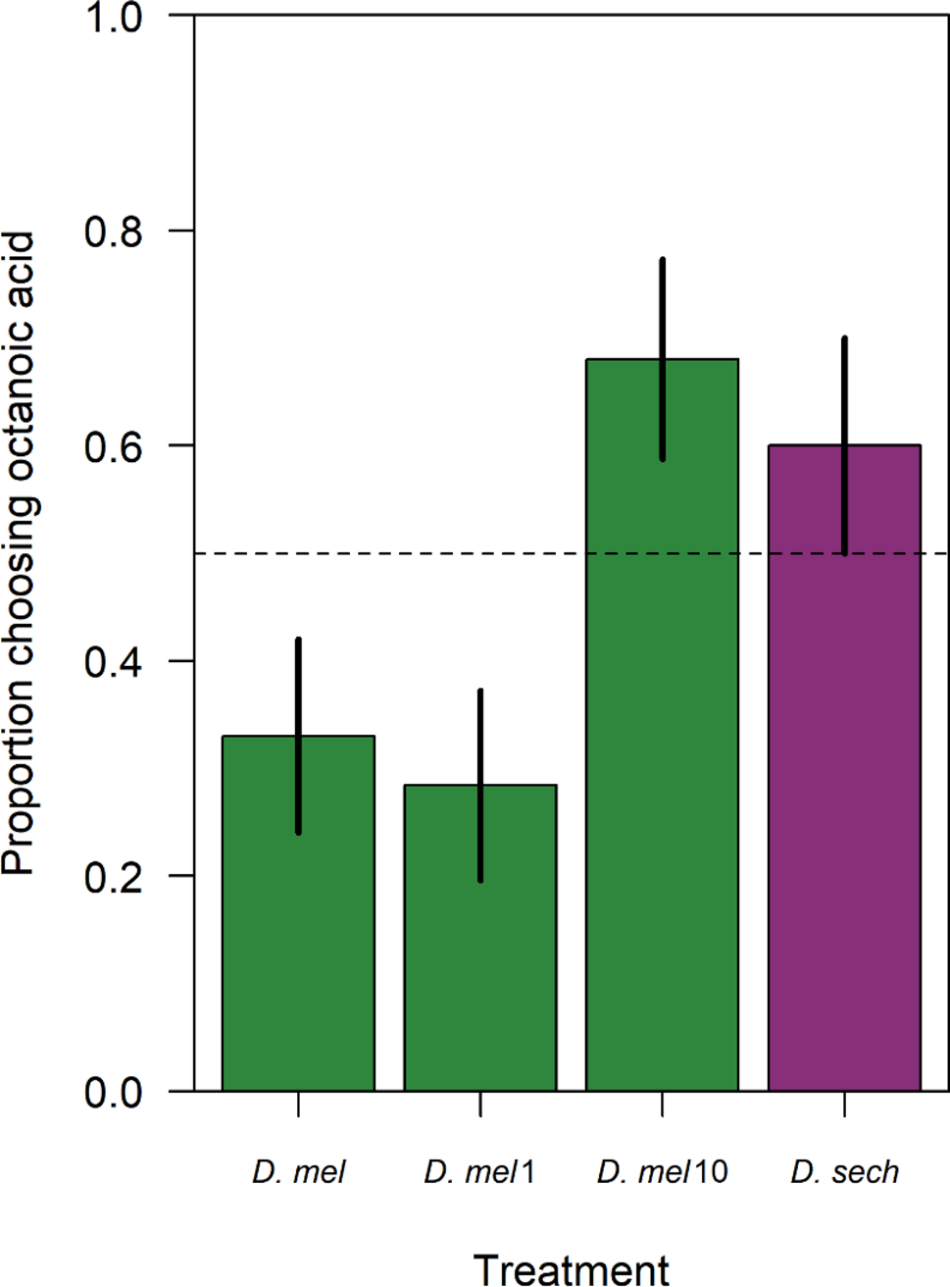
The proportion of flies that chose to disperse to fly medium containing octanoic acid versus regular fly food. The treatments include: *D. melanogaster* reared on regular fly food (*D. mel*), *D. melanogaster* reared on fly food supplemented with *D. sechellia* gut microbiota for one generation (*D. mel* 1), *D. melanogaster* reared on fly food supplemented with *D. sechellia* gut microbiota for 10 generations (*D. mel* 10) and *D. sechellia* (*D. sech*) reared on *M. citrifolia*. Sample means and 95% confidence intervals from 10,000 bootstrap simulations shown.

*D. melanogaster* is highly averse to the scent profile of octanoic acid^12^, compared to *D. sechellia* for which it is a chemoattractant^22^. In this study, model selection using AIC showed that variation in aversion to octanoic acid was best explained by the effect of treatment only (no effect of sex). *D. melanogaster* that hadn’t been exposed to *D. sechellia* gut microbiota, or had only been exposed to *D. sechellia* gut microbiota for one generation, were significantly more averse to octanoic acid than *D. sechellia* (Fig. 2: *z* = − 3.778, *p* < 0.001, and *z* = − 4.433, *p* < 0.001 respectively)*.* However, after 10 generations of being reared on food supplemented with *D. sechellia* gut microbiota, there was no difference in the aversion of *D. melanogaster* and *D. sechellia* to food containing octanoic acid (Fig. 2: *z* = 1.173, *p* = 0.241). Furthermore, after 10 generations, *D. melanogaster* displayed an active preference for food containing octanoic acid (proportion choosing food with octanoic acid > 0.5). Thus, we have shown that dietary exposure to gut microbes can cause individuals to evolve not only to tolerate, but display an active preference for food that was previously repulsive to them.

Highlighting the molecular/physiological mechanism that led to *D. melanogaster* evolving to prefer food containing octanoic acid over standard fly food is beyond the scope of this study. We make three suggestions as to the processes that may have led to the patterns observed in our data: (1) gut microbiota was maternally inherited, meaning *L. plantarum* accumulated in the gut over 10 generations, (2) incorporating *L. plantarum* into the gut provided a fitness advantage; thus, over 10 generations individuals evolved to harbour more *L. plantarum* in their guts, and (3) food preference is driven by *L. plantarum*. Prediction 2 seems unlikely given that, between choice trials, *D. melanogaster* were reared on standard food—meaning there would be no obvious fitness advantage to having increased amounts of *L. plantarum* in the gut. However, prediction 1 is plausible given that there is evidence to suggest that gut microbiota is a heritable trait in *D. melanogaster*^23^. Moreover, prediction 3 is also plausible, since gut microbes, like all organisms, are under selective pressure to increase their fitness. One way for gut microbes to increase fitness could be through the manipulation of the feeding behaviour of their host^24,25^. This could lead to hosts displaying a preference for food items that maximise the fitness of said microbes. The impact of a given host dietary preference on gut microbe fitness is likely to vary between microbe species. This potentially creates an evolutionary conflict between different species of microbe which may ultimately limit the impact that any one microbe species can have on host foraging behaviour. As such, Alcock et al.^24^ hypothesise that manipulation of host feeding behaviour by gut microbes is more likely to occur when gut microbiota diversity is low. It could be that the foreign gut microbiota ingested by *D. melanogaster* in our experiment displaced the original gut microbiota, reducing microbiome diversity thereby increasing potential for gut microbes to manipulate host feeding behaviour. The ability of gut microbes to manipulate host feeding behaviour has already been demonstrated empirically. For example, *Acetobacter spp*. and *Lactobacillus spp*. have been shown to alter *D. melanogaster* food preferences and foraging decisions, with flies whose gut microbiota was suppressed or mono-associated with one or the other of the bacteria demonstrating a shift in dietary preference^26^. This Alcock et al.^24^ hypothesis could explain why octanoic acid aversion was not expressed from the first generation but after ten generations of our gut microbiota manipulated *D. melanogaster*. Indeed, attraction/repulsion changes toward a food resource induced by gut microbiota is not expected until the bacteria population grows sufficiently^24,25^.

In conclusion, we have shown that the composition of an individual’s gut microbiota can be highly plastic in response to changing food resources. Also, we have shown that the ingestion of gut microbes from other species can lead to large changes in dietary preference over short evolutionary timescales. In an era where the spatial overlap of species is ever-increasing^27^, exposure to microbes from other species is becoming increasingly common^28,29^. As such, exposure to foreign microbes and the capacity for an individual’s gut microbiota to vary in response to changing food availability may become a potent evolutionary driver of dietary niche differentiation.

## Methods

We purchased three outbred lines of *D. sechellia* collected from Cousin Island (lines 0.21, 0.07 and 0.08), Seychelles in 1980 from the National Drosophila Species Stock Centre, formerly in San Diego (California, USA). Experimental *D. melanogaster* were wild-type *Wolbachia*-free stocks isolated from an outbred population collected in Lyon, France. All flies were kept in standard 75 × 25 mm *Drosophila* vials at 25 °C on a 12:12 h light–dark cycle and fed by a yeast/agar/maize/sugar (ASG) food medium [for 1 l of water: 85 g of sugar, 60 g of corn, 20 g of yeast, 10 g of agar and 25 ml of nipagin (100 g l^−1^)]. Flies were moved to new vials every 4 days.

### Manipulating diet in *D. sechellia*

Newly emerged adults during the nights were removed at 9 am every morning. Newly emerged adults during the day were collected at 12 p.m. and 5 p.m. to ensure they were virgin. Collected virgin adults from the three *D. sechellia* lines were transferred onto ASG where they remained for 1 week. Two male and two female adults from each of the three lines were then removed from the population for microbial analysis. The remaining flies were then transferred into new vials containing 25 g of *M. citrifolia*. After 1 week, two male and two female adults from each of the three lines were removed from the population for microbial analysis. The remaining flies were then transferred back to ASG where they remained for one more week before two male and two female adults were removed from the population for microbial analysis.

### Sequencing of gut microbiota

Collected flies were surface sterilised in 70% ethanol, rinsed in distilled water and air dried. The head was then removed and guts from two flies were dissected and isolated in Eppendorf tubes containing 250 μl of sterile Lysogeny Broth (LB)^30^. Gut tissue was homogenised with a sterile plastic pestle, and 100 μl of gut homogenate was pipetted onto BHI (Brain, Heart Infusion)^31^ agar before being spread-plated using a sterile glass loop. Plates were left to air dry aseptically, before being closed and sealed with parafilm for incubation at 25 °C for 72 h. Bacterial load was quantified by performing Colony Forming Unit (CFU) counts. Single colonies were isolated using a sterile 1 μl loop and placed into an Eppendorf with 10 μl sterile water before being analysed using PCR and Sanger sequencing, as described previously^32^. PCR amplification was performed in a 25 μl reaction volume consisting of 10 μl nuclease-free water, 13 μl Taq green master mix, 0.5 μl of forward primer 27F (5’- AGAGTTTGATCMTGGCTCAG-3’) and reverse primer 1492R (5′-GGTTACCTTGTTACGACTT-3’) and 1 μl of template DNA. Thermal cycling was performed for 90 s at 95 °C as initial denaturation, followed by 35 cycles of 30 s at 95 °C for denaturation, 30 s at 55 °C as annealing, 90 s at 72 °C for extension, and final extension at 72 °C for 5 min. 1500 bp 16S PCR products were purified with Ampure beads and subjected to Sanger sequencing. The resulting sequences were identified using NCBI BLAST against the nt database^33^.

### Exposure of *D. melanogaster* to *D. sechellia* gut microbiota

Using the procedure described above, we extracted gut solute from an equal number of male and female *D. sechellia* reared on *M. citrifolia* and evenly applied 30 µL of gut solute to the surface of ASG and left to dry for 20 min. Newly emerged *D. melanogaster* virgin flies from the stock population were collected and placed into vials containing the gut solute at a constant density of 10 males and 10 females per vial. After pupation, adults were removed to prevent them breeding with offspring. When new adult flies emerged, they were placed into a new vial containing the same ASG and 30 µL gut solute mix. This process was repeated for 10 generations. At generations 1 and 10, a sample of the population were removed (generation 1: *n*_*male*_ = 50, *n*_*female*_ = 51; generation 10: *n*_*male*_ = 48, *n*_*female*_ = 49) for use in the aversion trials. The introduction of the ASG/gut solute diet was staggered such that aversion trials using individuals from the generation 1 treatment could be run simultaneously with trials using individuals from the generation 10 treatment.

It should be noted that conventional *D. melanogaster* (harbouring a microbiota) were used and not axenic (which would lack a microbiota) flies. Indeed, axenic *D. melanogaster* are known to have altered physiology in terms of weight and egg to adult survival but also behaviours, notably reduced locomotion in females^32^. Therefore, since aversion assays inherently involve movement towards a food/patch source, conventional flies were preferred. Thus, our experiment is likely to be informative of how food preferences in wild *D. melanogaster* change in response to exposure to novel gut microbes.

### Aversion to octanoic acid

Flies used in the aversion trials were separated by sex from emergence to prevent mating and maintained in vials containing ASG for 3 days prior to experimentation. Aversion trials were performed using a similar methodology to that utilised previously^34^. Flies were moved to individual petri dishes (100 mm diameter and 15 mm height) containing 10 g of ASG at either end with a marked line half-way across clearly showing the two separate sides. On one side of the petri dish, 10 µL of ≥ 99% octanoic acid (Sigma-Aldrich) was added to the ASG. After 5 min, we recorded the food source that the fly had settled on as the fly’s choice, flies that did not choose a food source after 5 min were not recorded.

### Data analysis

The abundance of Colony Forming Units (CFU) was analysed using a generalised linear mixed effects model. Our maximal model included an interaction between dietary treatment and bacterial species as a fixed effect along with sex. Because several CFU readings were taken from single individuals, a random intercept for individual was included in the model. So that the best-fitting model could be selected, the distribution of the residual error was compared between models using logged and non-logged response data. Data from the aversion trials was analysed using a generalised linear model with treatment and sex as fixed effects and binomial error correction. The minimum adequate model for both analyses was determined using Akaike’s information Criterion (AIC). Following the reasoning of Arnold (2010)^35^, fixed effects that changed the AIC score by < 2 were removed from the model. All data was analysed in R version 3.6.1 (2019)^36^. The mixed effects model and associated *p*-values were generated using the ‘lme4’ and ‘lmerTest’ packages^37,38^.

## Supplementary Information

Supplementary Information.

## Data Availability

All data related to bacterial counts are presented in Table 1 in the supplementary material. Raw data and analysis code related to the aversion trials can be found at: 10.5281/zenodo.5179855.
