# Supplementary material for: Exposure to foreign gut microbiota can facilitate rapid dietary shifts
Source: Sci Rep. 2021 Aug 18;11:16791. doi: 10.1038/s41598-021-96324-5 (PMC8373899; doi:10.1038/s41598-021-96324-5)
Supplement: Supplementary file 1 — Supplementary Information. [file 41598_2021_96324_MOESM1_ESM.pdf]

Exposure to foreign gut microbiota can facilitate rapid dietary shifts

Heys C[1,2], Fisher AM[1*], Dewhurst AD[1], Lewis Z[3] and Lizé A[1,4]

[1]Department of Evolution, Ecology and Behaviour, University of Liverpool, Liverpool, L69 7ZB, UK

[2]School of Life Sciences and Education, Staffordshire University, Stoke-on-Trent, ST4 2RU, UK

[3]School of Life Sciences, University of Liverpool, L69 7ZB, UK

[4]UMR CNRS 6553 ECOBIO, University of Rennes 1, 35042, Rennes, France

*Corresponding authors: adam.fisher@liverpool.ac.uk

Supplementary material

Table 1 - Number of bacterial colonies isolated from the midgut of adult *D. sechellia*. Flies were first reared on ASG (represented by ASG 1), then moved onto noni (*M. citrifolia*), before being transferred back onto ASG (ASG 2).

Diet	Strain	Replicate	Sex	*L. plantarum*	*Paenibacillus sp.*	*B. cereus*
ASG 1	0.21	1	F	3.12x10^2	0.20x10^1	0
ASG 1	0.21	2	F	1.82x10^2	1.40x10^1	0
ASG 1	0.21	1	M	1.58x10^2	0	0.10x10^1
ASG 1	0.21	2	M	2.50x10^1	0.90x10^1	0
ASG 1	0.07	1	F	5.11x10^3	1.81x10^2	0.50x10^1
ASG 1	0.07	2	F	5.94x10^3	1.23x10^2	0.20x10^1
ASG 1	0.07	1	M	4.88x10^3	5.40x10^1	0.70x10^1
ASG 1	0.07	2	M	3.58x10^3	1.75x10^1	2.20x10^1
ASG 1	0.08	1	F	6.25x10^3	2.02x10^2	2.70x10^1
ASG 1	0.08	2	F	4.09x10^3	1.96x10^2	1.50x10^1
ASG 1	0.08	1	M	3.17x10^3	2.70x10^1	0.40x10^1
ASG 1	0.08	2	M	2.89x10^3	8.80x10^1	0
Noni	0.21	1	F	2.72x10^3	0	0
Noni	0.21	2	F	1.78x10^3	0	0
Noni	0.21	1	M	1.62x10^2	0	0
Noni	0.21	2	M	1.59x10^2	0	0
Noni	0.07	1	F	1.43x10^3	0	0
Noni	0.07	2	F	1.34x10^3	0	0
Noni	0.07	1	M	2.55x10^2	1.50x10^1	0
Noni	0.07	2	M	1.92x10^2	0.70x10^1	0
Noni	0.08	1	F	4.5x10^2	0	0
Noni	0.08	2	F	1.81x10^2	0	0
Noni	0.08	1	M	8.10x10^1	0.50x10^1	0
Noni	0.08	2	M	7.20x10^1	0	0
ASG 2	0.21	1	F	1.21x10^3	2.40x10^1	0
ASG 2	0.21	2	F	1.45x10^3	1.20x10^1	0.10x10^1
ASG 2	0.21	1	M	1.22x10^2	0.20x10^1	0.10x10^1
ASG 2	0.21	2	M	2.31x10^2	0.50x10^1	0.20x10^1
ASG 2	0.07	1	F	4.51x10^2	1.50x10^2	0.70x10^1
ASG 2	0.07	2	F	5.22x10^2	8.90x10^2	0.20x10^1
ASG 2	0.07	1	M	2.09x10^3	2.90x10^1	0.60x10^1
ASG 2	0.07	2	M	2.87x10^3	4.60x10^1	1.30x10^1
ASG 2	0.08	1	F	3.22x10^3	1.98x10^2	2.90x10^1
ASG 2	0.08	2	F	2.46x10^3	2.51x10^2	2.20x10^1
ASG 2	0.08	1	M	1.78x10^3	1.12x10^2	0.50x10^1
ASG 2	0.08	2	M	2.34x10^3	1.43x10^2	0.70x10^1
